# Cell Wall Protein 2 as a Vaccine Candidate Protects Mice Against *Clostridioides difficile* Infection

**DOI:** 10.3390/vaccines13010021

**Published:** 2024-12-30

**Authors:** Shaohui Wang, Joshua Heuler, Jessica Bullock, Junling Qin, Soumyadeep Chakraborty, Agbendeh Lubem Nathaniel, Shifeng Wang, Xingmin Sun

**Affiliations:** 1 Department of Molecular Medicine, Morsani College of Medicine, University of South Florida, Tampa, FL 33620, USA; 2 Department of Chemistry, University of South Florida, Tampa, FL 33620, USA; 3 Department of Infectious Diseases and Immunology, College of Veterinary Medicine, University of Florida, Gainesville, FL 33620, USA

**Keywords:** *Clostridioides difficile* infection (CDI), colonization, Cwp2, vaccine

## Abstract

Background/Objectives: *Clostridioides difficile* is a Gram-positive, spore-forming enteric pathogen that causes intestinal disorders, including inflammation and diarrhea, primarily through toxin production. Standard treatment options for *C. difficile* infection (CDI) involve a limited selection of antibiotics that are not fully effective, leading to high recurrence rates. Vaccination presents a promising strategy for preventing both CDI and its recurrence. Cell wall protein 2 (Cwp2), a highly immunogenic and abundant surface-exposed *C. difficile* cell wall protein, plays an important role in the bacterium’s adherence in vitro. In this study, we aimed to analyze the homology and immunogenicity of Cwp2 and its protection efficacy as a vaccine candidate against CDI in mice. Methods: we conducted in silico analyses to assess the homology and immunogenicity of Cwp2, and we evaluated its potential as a vaccine candidate against CDI using a mouse model of immunization and infection. Results: Our in silico analyses predicted the immunogenic region (functional domain) of Cwp2 and revealed its high homology among various toxinotypes and ribotypes (R.T.s) or sequence types (S.T.s). Immunizations of mice with the Cwp2 functional domain (Cwp2_A) induced potent IgG/A antibody responses against Cwp2_A, protected mice from CDI, and reduced *C. difficile* spore and toxin levels in feces post-infection. Additionally, anti-Cwp2_A sera inhibited the binding of *C. difficile* vegetative cells to HCT8 cells. Conclusions: Our report demonstrates for the first time the potential of Cwp2_A as an effective vaccine candidate against CDI in mice.

## 1. Introduction

*Clostridioides difficile* is a Gram-positive, spore-forming enteric pathogen that causes a range of intestinal disorders and that is a significant public health concern worldwide. Symptoms of *C. difficile* infection (CDI) can vary from diarrhea and intestinal inflammation to pseudomembranous colitis and even death [1,2]. The commonly used treatments for CDI, which include a limited number of antibiotics such as fidaxomicin, vancomycin, and metronidazole [3,4,5,6], are not fully effective and are often associated with high recurrence rates (15–35%) [7,8,9]. Active vaccination offers a promising approach for preventing CDI and its recurrence [10]; however, no vaccine against CDI has been licensed to date [11,12,13].

The symptoms of CDI are primarily caused by two major *C. difficile* toxins, toxin A (TcdA) and toxin B (TcdB) [14,15], while binary toxin (CDT) produced by about 20% of *C. difficile* strains may also play a minor role in the pathogenesis of *C. difficile* [16,17]. Consequently, significant efforts have been made to develop vaccines targeting TcdA/TcdB through parenteral immunizations [18,19,20], including two vaccine candidates in clinical trials: VLA84 and the genetically modified TcdA and TcdB from Pfizer [11]. However, given that *C. difficile* is an enteric pathogen with a high rate of infection recurrence, effective vaccines should not only target the toxins but should also block *C. difficile* colonization. To address this, we focused on identifying novel *C. difficile* surface colonization and adhesion factors that could be explored as potential components of an effective vaccine against CDI and its recurrence.

**Figure 1:**
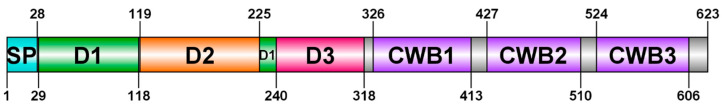
**Domain architecture of cell wall protein 2 (Cwp2) (WP_009891054.1) from *C. difficile* R20291.** The signal peptide (SP) is followed by the functional region, which includes domain 1 (D1), domain 2 (D2), and domain 3 (D3); D2 is connected to D3 via a strand of 13 aa in D1. The cell wall binding domain (CWB) has 3 repeated regions, CWB1, CWB2, and CWB3, as indicated in UniProt. The schematic representation of the domain architecture was developed in DOG 2.0.

The surface layer (S-layer) of *C. difficile* contains over 30 proteins, with the majority of the S-layer being formed by SlpA, which is composed of low- and high-molecular weight S-layer proteins (LMW SLP and HMW SLP) [21,22]. The *cwp2* gene is found within the *slpA* locus, part of a cluster of S-layer-associated genes surrounding *slpA* [23,24]. Cell wall protein 2 (Cwp2) contains a functional region (Cwp2_A) that includes domains 1, 2, and 3, as well as three cell wall binding domains (CWB1, CWB2, and CWB3) [25] (Figure 1), a conserved feature for *C. difficile* S-layer proteins [26,27]. Cwp2 is recognized by nearly all CDI patient sera [28], indicating it as highly immunogenic. Additionally, a *cwp2* knockout mutant in *C. difficile* strain 630 leads to increased TcdA release and impaired cellular adherence in vitro [25]. Cwp2 is highly and constitutively expressed during normal *C. difficile* growth [29] and is also found in the *C. difficile* spore coat [30]. In this study, we aimed to analyze the homology and immunogenicity of Cwp2 and its protection efficacy as a vaccine candidate against CDI in mice.

## 2. Materials and Methods

### 2.1. Phylogeny and Homology Analysis of Cwp2

*C. difficile* strains were sourced from public databases and previous studies (Supplementary Table S1) to create a dataset of strains from different ribotypes. The genomes of each strain were obtained from either GenBank (National Center for Biotechnology Information) or the *Clostridioides difficile* genome database from Enterobase [31]. After mining the amino acid sequences for Cwp2 from each genome, we performed MUSCLE alignments of the sequences using MegaX (version 11.0.13) [32] set to default settings. Following sequence alignment, we constructed maximum likelihood phylogenetic trees with 100 bootstrap replicates, also using MegaX. The cluster patterns from these phylogenetic trees guided the selection of specific Cwp2 sequences for domain analysis. The selected Cwp2 sequences were then aligned once more using the MUSCLE algorithm through the MPI Bioinformatics Toolkit server [33,34], and then the output files were visualized in Jalview (version 2.11.4.1) [35].

### 2.2. Expression and Purification of Recombinant Protein Cwp2_A

The functional domain Cwp2_A (amino acids 29–318) of the *cwp2* gene from *Clostridioides difficile* R20291 was PCR-amplified and cloned into the NheI and XhoI sites of the expression vector pET28a in *E. coli* DH5α. The forward primer sequence was 5′-agatgctagccaggtaaaaaaagaaacaataac-3′, and the reverse primer was 5′-ggtgctcgagttattctaatgcagctttggcat-3′. The Cwp2_A protein fragment, containing an N-terminal His-tag, was purified using Ni-affinity chromatography [36]. Briefly, the BL21 culture pellet (from a 1000 mL culture) was resuspended in 40 mL of lysis buffer (300 mM NaCl, 20 mM imidazole, 20 mM NaH_2_PO_4_, 500 μM EDTA, protease inhibitor cocktail; Cat #P8849, Sigma), adjusted to pH 8.0. Cells were disrupted via sonication, and the lysate was centrifuged at 15,000× *g* for 20 min. The resulting supernatant was passed through a nickel-charged HiTrap chelating HP column (Amersham Biosciences, Piscataway, NJ, USA). The bound His-tagged Cwp2_A protein was eluted using a buffer containing 250 mM imidazole, 300 mM NaCl, and 20 mM NaH_2_PO_4_, pH 8.0. The eluted protein was subsequently desalted and further purified using a HiTrap Q column (Amersham Biosciences), where Cwp2_A was eluted via a NaCl gradient. Protein purity was confirmed by an SDS-PAGE analysis, and the purified Cwp2_A protein was stored at −80 °C until use.

### 2.3. Preparation of C. difficile Spores

Sporulation of the *C. difficile* R20291 strain was induced in Clospore medium, and spores were purified as described previously [36,37,38].

### 2.4. Mouse Immunization and Mouse Model of CDI

All studies were conducted in accordance with the *Guide for the Care and Use of Laboratory Animals* of the National Institutes of Health and were approved by the Institutional Animal Care and Use Committee (IACUC) at the University of South Florida (Protocol #IS00011981). Wild-type C57BL/6 mice were obtained from Charles River Laboratories and housed under standardized conditions in a specific pathogen-free (SPF) environment. Mice were maintained on a semi-natural light cycle of 14 h light and 10 h dark.

The Cwp2_A protein was treated with endotoxin removal resin according to the manufacturer’s instructions (Cat# P188274, Thermo Scientific, Waltham, MA, USA) prior to immunization. For animal immunization experiments, Cwp2_A (10 or 20 μg) was administered in PBS with aluminum (Imject Alum, cat# 77161, Thermo Scientific, Waltham, MA, USA) as an adjuvant. Thoroughly mixed Imject Alum was added dropwise to Cwp2_A solution while mixing so that the final volume ratio of Imject Alum to Cwp2_A was 1:2 (e.g., 100 μL of Imject Alum was added to 200 μL of immunogen); the mixture was continuously mixed for 30 min after adding Imject Alum.

Mice (*n* = 10, with 5 males and 5 females, 6 weeks old) were immunized three times at 12-day intervals via intraperitoneal (i.p.) injection, as described previously [37,39]. Control mice received equivalent volumes of PBS. Sera and fecal samples were collected. Immunization experiments were repeated once. Seven days after the third immunization, both immunized and control mice were provided with drinking water containing a mixture of five antibiotics: kanamycin (40 mg/kg), gentamycin (3.5 mg/kg), colistin (4.2 mg/kg), metronidazole (21.5 mg/kg), and vancomycin (4.5 mg/kg) for four days. This was followed by two days of autoclaved water. Mice then received a single intraperitoneal injection of clindamycin (10 mg/kg) prior to being challenged with 10⁶ *C. difficile* R20291 spores per mouse via oral gavage, as described previously [40]. Post-infection, mice were monitored daily for one week to assess survival, weight changes, diarrhea, and other disease symptoms. Diarrhea was defined by the presence of wet tails and loose or watery feces. Mouse mortality was recorded as the number of mice that died post-infection or that were euthanized due to weight loss exceeding 20%.

### 2.5. ELISA for Anti-Cwp2_A IgG/A

An ELISA analysis was performed as described previously [41]. Briefly, Costar 96-well ELISA plates were coated with 100 µL/well of Cwp2_A (0.5 µg/mL) and incubated overnight at 4 °C. Unbound material was washed off, and the wells were blocked with 300 µL of blocking buffer (PBS + 5% dry milk) for 2 h at room temperature. Subsequently, 100 µL of 10-fold diluted sera or fecal samples were added to each well and incubated for 1.5 h at room temperature. After washing with PBS, 100 µL of either HRP-conjugated mouse IgG (1:3000) or IgA (1:3000) was added and incubated for 30 min to 1 h. Following another PBS wash, TMB substrate was added to induce color development for 5–30 min at room temperature. The reaction was terminated by adding H_2_SO_4_, and the optical density (OD) at 450 nm was measured using a spectrophotometer. The anti-Cwp2_A IgG/A titer for each sample was defined as the dilution factor at which the OD_450_nm was at least 2-fold higher than that of serum samples from non-immunized mice.

### 2.6. Quantification of C. difficile Spores in Mouse Feces [37]

Fecal samples were collected on days 0, 1, 3, 5, and 7 post-infection. Fifty milligrams of feces were suspended in 500 µL of sterile water and incubated at 4 °C for 16 h. To eliminate vegetative cells, the samples were treated with 500 µL of absolute ethanol (Sigma-Aldrich, St. Louis, MO, USA) for 1 h at room temperature. After vortexing, the samples were serially diluted and plated onto BHI medium supplemented with taurocholate (0.1% *w*/*v*), cefoxitin (8 µg/mL), and D-cycloserine (250 µg/mL). Plates were incubated anaerobically at 37 °C for 48 h, and colonies were subsequently counted. The results were expressed as CFU per gram of feces.

### 2.7. ELISA for TcdA and TcdB [37]

Following the challenge with *Clostridioides difficile* spores, fecal samples were collected and dissolved in PBS (0.1 g/mL) containing a protease inhibitor cocktail. The samples were centrifuged, and the resulting supernatants were collected to determine TcdA and TcdB concentrations using ELISA. Briefly, 96-well Costar microplates were coated overnight at 4 °C with 100 µL of anti-TcdA and anti-TcdB antibodies (1 µg/mL each) prepared in phosphate-buffered saline (PBS). The following day, wells were blocked with 300 µL of blocking buffer (PBS + 5% dry milk) for 2 h at room temperature. Standards and samples (100 µL each) were added in duplicate and incubated for 90 min at 25 °C. After washing, HRP-conjugated chicken anti-*C. difficile* TcdA/TcdB antibodies (1:5000 dilution in PBS; Gallus Immunotech, Shirley, MA, USA) were applied for 30 min at room temperature. Following a final wash, TMB microwell peroxidase substrate was added and incubated for 20 min at room temperature in the dark. The reaction was stopped by adding 2 N H_2_SO_4_, and absorbance was measured at 450 nm using a plate reader.

### 2.8. Adherence Inhibition Assays

The adherence of *Clostridioides difficile* R20291 vegetative cells to human gut epithelial cells was evaluated as previously described [37,42]. Briefly, HCT-8 cells were cultured to 95% confluence (1 × 10^5^ cells/well) in a 24-well plate and transferred to an anaerobic chamber. The cells were infected with 1.5 × 10^6^ log-phase R20291 vegetative cells at a multiplicity of infection (MOI) of 15:1, followed by incubation at 37 °C for 100 min. Prior to infection, R20291 vegetative cells were preincubated with anti-Cwp2_A sera at dilutions of 1/50, 1/100, and 1/200 for 30 min. Following incubation, the cell-R20291 mixture was washed three times with 1× PBS by centrifugation at 800× *g* for 1 min to remove non-adherent cells. Supernatants from each wash step were collected, and non-adherent R20291 cells were enumerated on pre-reduced BHI agar. Control experiments included R20291 cells incubated with PBS or pre-immune sera (1/25 dilution). Adhesion assays were performed in triplicate. The percentage of R20291 adherence was calculated using the formula: adherence (%) = 100 × (initial CFU/mL − eluted CFU/mL)/initial CFU/mL.

### 2.9. Determination of Cytokine Expression by Real-Time PCR

Mice spleens (*n* = 5) were obtained from Cwp2-immunized and control (non-immunized) mice 13 days after the second immunization. Splenocytes were isolated through 70 µm and 40 µm cell strainers using syringe plunges to prepare single cell suspensions. Cells were counted and seeded in RPMI 1640 medium (Thermofisher, Waltham, MA, USA) containing 10% FBS and incubated at 37 °C and 5% CO_2_. The cells were then pulsed with 10 µg/mL Cwp2 for 72 h and used for the flow cytometry analysis below. A portion of immunogen-pulsed cells were collected for the expression analysis of IL-4, IL-5, IL-17, IFN-γ, and TNF-α by real-time PCR. The primers used (Table 1) are from published papers [43,44,45]. The GAPDH gene was chosen as the reference for internal standardization. GAPDH is a well-accepted housekeeping gene, which has been used in other studies [43,45].

A real-time PCR analysis was performed on a CFX Opus 96 real-time PCR system (Bio Rad, Hercules, CA, USA). SYBR Green/ROX qPCR Master mix (Thermo Fisher) was used according to the manufacturer’s protocol. The reaction conditions were as follows: 95 °C for 10 sec, followed by 40 cycles of 95 °C for 15 s, and 60 °C for 30 sec. The reaction of each sample was performed in triplicate. A dissociation analysis was performed at the end of each PCR reaction to confirm the amplification specificity. After the PCR program, data were analyzed and quantified with the comparative Ct method (2^–ΔΔCt^) based on Ct values for cytokine genes and GAPDH in order to calculate the relative mRNA expression level.

### 2.10. Antigen-Specific T Cells Proliferation Assays

The spleen cells collected above were pulsed with 10 µg/mL Cwp2 for 72 h. The cells were stained by antibodies against CD3, CD4, and CD8 (BioLegend, San Diego, CA, USA) by a flow cytometry analysis.

### 2.11. Statistical Analysis

Animal survival was assessed using Kaplan–Meier survival analysis, with statistical significance being determined using the log-rank test. For comparisons between two groups, the non-parametric Student’s *t*-test was employed, while one-way analysis of variance (ANOVA) with post hoc Bonferroni tests were used for comparisons involving more than two groups. The results are presented as the means ± standard error of the mean (SEM), and differences were considered statistically significant at *p* < 0.05 (*). All statistical analyses were conducted using GraphPad Prism 6 software. Non-parametric tests were chosen because the data did not follow a normal distribution.

## 3. Results

### 3.1. The Functional Domain of Cwp2 Is Highly Immunogenic Based on B Cell Epitope Analysis

**Figure 2:**
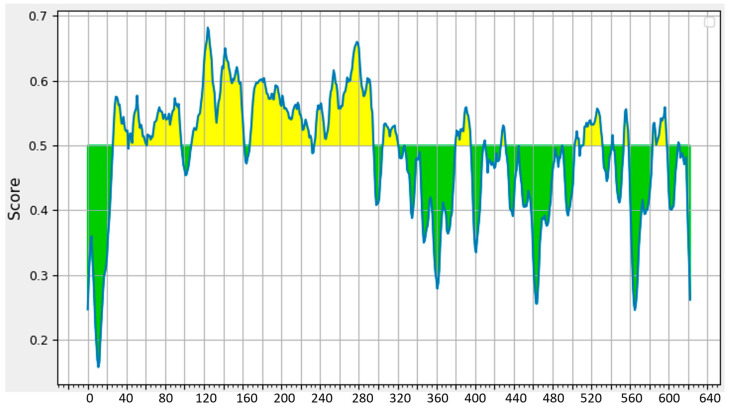
**Predicted immunogenic regions (in yellow) of Cwp2.** B cell epitopes of Cwp2 were predicted using the BepiPred-2.0 server (https://www.iedb.org/; accessed on 6 May 2023). The residues with scores above the threshold (default value is 0.5) are predicted to be part of an epitope and are colored in yellow on the graph (where the Y-axis depicts residue scores and the X-axis depicts residue positions in the sequence).

To assess immunogenicity, we conducted a B cell epitope analysis using the BepiPred-2.0 server (https://www.iedb.org/; access date 6 May 2023). The functional domain of Cwp2 (aa 27-295, Cwp2_A), which includes domains 1, 2, and 3, contains the majority of immunogenic peptides (highlighted in yellow). In contrast, the cell wall binding regions (CWB1, CWB2, and CWB3) have very few low-immunogenic peptides. This indicates that Cwp2_A is potentially highly immunogenic (Figure 2).

### 3.2. Phylogeny and Homology of Cwp2 Across C. difficile Strains from Various Toxinotypes and Ribotypes

**Figure 3:**
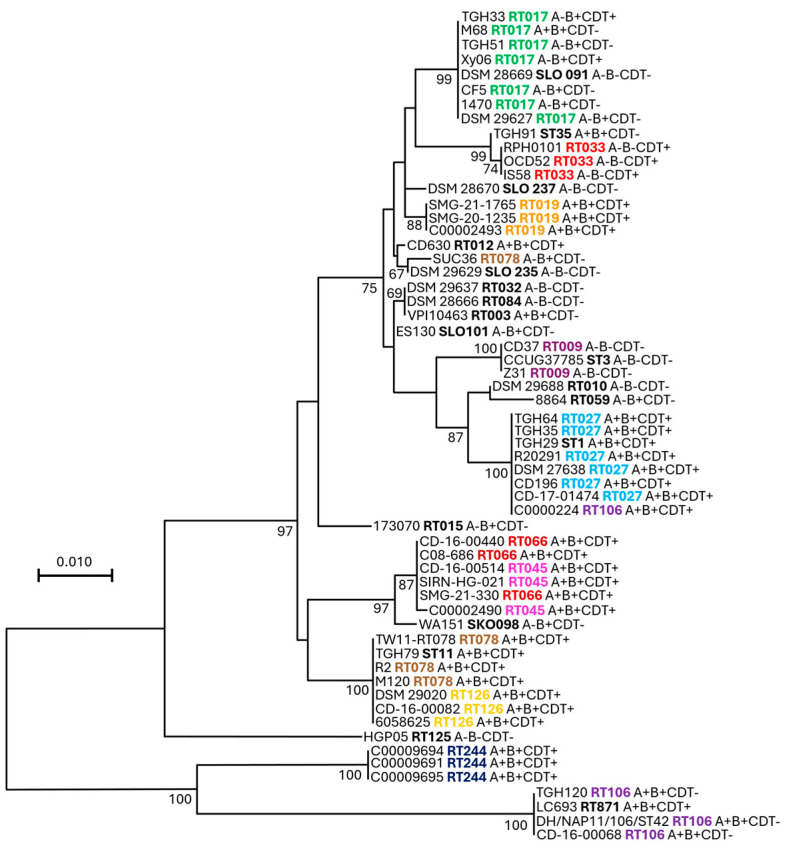
**Cwp2 phylogeny.** Amino acid sequences of Cwp2 were aligned with the MUSCLE algorithm in MegaX before computing a maximum likelihood tree with 100 bootstrap replicates (bootstrap values >50 are displayed). Scale bars indicate 0.010 substitutions per site. The ribotype (or sequence type) of each source strain is displayed adjacent to the strain name. Ribotypes with multiple representatives have multicolored labels, while black labels indicate ribotypes with only one representative on the tree. Toxinotypes for each strain are indicated to the right of the ribotype for each strain.

To identify effective vaccine candidates against CDI, immunogens should be conserved across different *C. difficile* strains. To this end, we investigated the phylogeny and homology of the Cwp2 protein in major toxinotypes as well as ribotypes (R.T.s) and sequence types (S.T.s). Maximum likelihood phylogenetic trees were generated using Cwp2 amino acid sequences from various *C. difficile* strains (Supplementary Table S1) to determine any correlation between sequence similarity and either the source strain’s R.T. or toxinotype (Figure 3). Our analysis revealed a strong correlation between *C. difficile* R.T. and Cwp2 relatedness. Most strains within a given R.T. have identical Cwp2 sequences (e.g., RT017, RT033, RT027). Only three ribotypes (RT106, RT045, RT078) do not have identical Cwp2 sequences, but the Cwp2 sequences within these ribotypes generally cluster close together (e.g., three out of four Cwp2 sequences for either RT016 or RT078 cluster together). For RT106 strains, strain C00000224 encodes a Cwp2 identical to those found in RT027 strains, while the other two RT106 Cwp2 sequences cluster in a different branch of the tree. In RT045 strains, C00002490 Cwp2 has aspartic acid (D) instead of asparagine (N) at position 214 compared with the other two RT045 Cwp2 sequences. Additionally, ribotype may indicate a lack of Cwp2, as all three RT023 strains examined (SIRN_ST-001, CD-16-00530, CD-15-00694) did not encode Cwp2, consistent with previous reports that Cwp2 is absent in some *C. difficile* strains [46]. Unlike the correlation observed between Cwp2 and ribotype, *C. difficile* toxinotype does not show a strong correlation with Cwp2 phylogeny.

**Figure 4:**
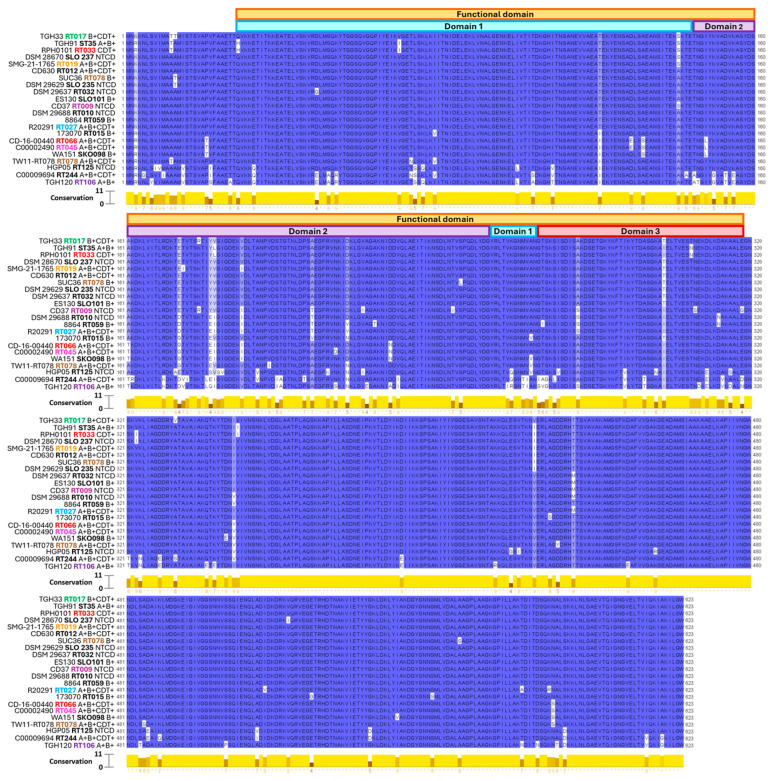
**Cwp2 homology.** Cwp2 sequences were aligned with MUSCLE and visualized with Jalview. The Jalview-calculated conservation scores are reported below the alignment from 0 (no conservation) to 11 (identical sequences). The ribotype of each source strain is displayed adjacent to the strain name and are color-coded for easier identification. The conserved amino acid sequences are highlighted in blue.

To more thoroughly examine Cwp2 sequence diversity, we aligned the sequences of the whole Cwp2 protein (Figure 4) using MUSCLE and visualized the output in Jalview (version 2.11.4.1) software. Representative sequences were selected from each cluster identified in the phylogenetic trees (Figure 3), ensuring that all non-identical sequences were included in the analysis. Upon aligning and visualizing twenty-two selected Cwp2 sequences, we found that they share 79% identical residues (130 out of 623) with a pairwise homology level ranging from 89% to 100% (Figure 4). Among the non-identical residues, 59% of the variations were located in the functional region (aa 23-318) [25] despite this region comprising only 46% of the protein. The pairwise homology level within the functional region ranges from 84% to 100%, while the remaining regions show a pairwise homology range of 93% to 100%. The functional region of Cwp2 is predicted to mediate the adhesive properties of the protein. Similarly, other Cwp proteins encoded by the *SlpA* locus, such as Cwp6, Cwp8, and LMW SLP, utilize their functional region for adhesion, and these surface proteins display significant inter-strain variability within their functional regions.

Based on structural similarities with Cwp8, prior studies argue that domain 2 is surface-exposed while domains 1 and 3 are located within the outer *C. difficile* membrane [47]. Because Cwp8 domain 2 is known to be the most variable domain within the protein, the same is hypothesized for Cwp2 [25]. In both cases, this variability is thought to facilitate immune evasion [25]. In our analysis, domain 2 has a greater total number of non-identical residues between the strains analyzed (36) when compared with domain 1 (23) or domain 3 (18). However, the ratio of non-identical residues to domain length is lowest in domain 2 (36/399, or 9%) compared with domain 1 (23/128, 18%) or domain 3 (18/53, or 34%).

Cwp2 also possesses the trimeric cell wall-anchoring CWB domains (CWB1, CWB2, and CWB3), which are a shared feature of *C. difficile* S-layer proteins [46]. Deletions of CWB2 prevent the anchoring of Cwp2 to the exterior of *C. difficile*, and Cwp2 also requires certain sequence patterns for correct anchoring as well [26]. We found that the trimeric CWB2 domains from Cwp6 and Cwp8 show moderate similarity (56 and 66% positives, respectively, see Table 1) with the C-terminal region of Cwp2 following the functional domain (amino acids 322-621). In Figure 4, 13% (41/305) of residue variations between the strains are found in the approximate region of the CWB2 domains (following the functional region) despite this region comprising about 49% of the protein length.

In summary, Cwp2 is present in all toxinotypes of *C. difficile* strains that we analyzed and is found in most ribotypes or sequence types, particularly in major clinically relevant ones (e.g., RT027, RT078, RT106, RT017). The Cwp2 amino acid sequences are generally identical within each ribotype, with a homology level of 89–100% among all ribotypes and sequence types analyzed. The functional domain of Cwp2 is also highly conserved, though it exhibits more variability compared with the cell wall binding (CWB) regions.

### 3.3. Immunization of Mice with Cwp2 Functional Domain (Cwp2_A) Induces Significant Anti-Cwp2_A Antibody Responses in Mice and Provides Protection Against C. difficile Infection

**Figure 5:**
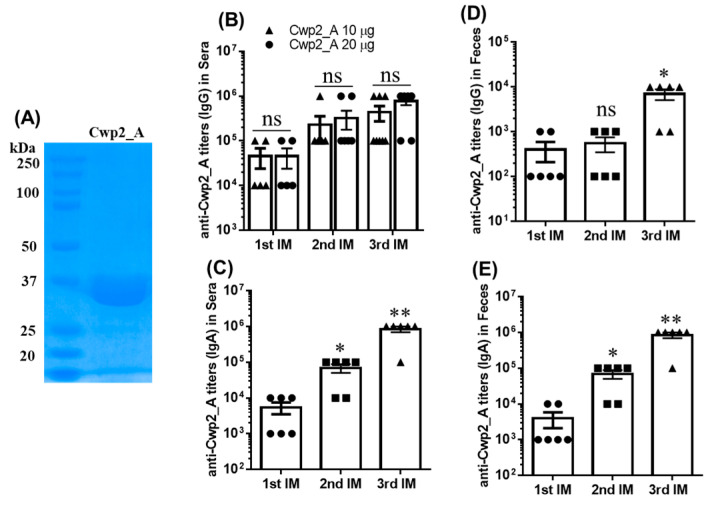
(**A**) Expression and purification of Cwp2_A. Cwp2_A was cloned in *E. Coli* BL21, and the protein was purified and analyzed on SDS-PAGE. (**B**) Immunization with Cwp2_A via the intraperitoneal (i.p.) route elicited anti-Cwp2_A antibody responses. Groups of mice (*n* = 5–8) were immunized three times with 10 µg or 20 µg of Cwp2_A with aluminum. In (**C**–**E**), 20 µg of protein was used. Anti-Cwp2_A IgG/IgA titers in sera and feces were determined using an ELISA analysis. Data are presented as the mean ± SEM (* *p* < 0.05; ** *p* < 0.01; ns, not significant; 2nd and 3rd IM vs. 1st IM in (**C**–**E**)).

Given that the Cwp2_A region is predicted to be highly immunogenic and moderately conserved, we hypothesized that Cwp2_A could serve as a potential vaccine component. To explore this, recombinant Cwp2_A with a 6xHis-tag was expressed in *E. coli* BL21 and purified to over 95% purity using Ni affinity chromatography (Figure 5A). Immunization of mice with 10 µg or 20 µg of Cwp2_A, combined with aluminum as an adjuvant and administered via the intraperitoneal route, induced high levels of IgG and IgA antibody responses against Cwp2_A in both sera and feces (Figure 5B–E).

**Figure 6:**
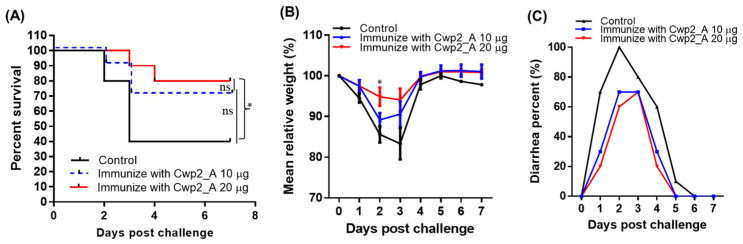
**Immunization with Cwp2_A provides mice significant protection against infection with *C. difficile*.** Immunized mice or controls (non-immunized mice) (*n* = 10) were challenged with *C. difficile* R20291 spores (10^6^/mouse). Survivals (**A**), weight changes (**B**), and diarrhea percentages (**C**) are shown. Data are presented as the mean ± SEM (ns, not significant; * *p* < 0.05; in (B), immunization with 20 µg vs. control).

The protective efficacy of Cwp2_A immunization was further evaluated in a mouse model of CDI. Mice were immunized three times with either 10 µg or 20 µg of Cwp2_A at 12-day intervals and subsequently challenged with 10^6^ spores of *C. difficile* R20291, a hypervirulent ribotype 027 strain. In the vehicle (PBS)-immunized group, significant disease symptoms were observed in all mice, including weight loss (Figure 6B) and severe diarrhea (Figure 6C), with approximately 60% succumbing by day 4 (Figure 6A). In contrast, Cwp2_A-immunized mice exhibited milder disease symptoms, characterized by reduced weight loss (Figure 6B) and lower rates of diarrhea (Figure 6C), along with higher survival rates (70% for the 10 µg Cwp2_A group and 80% for the 20 µg Cwp2_A group; Figure 6A).

**Figure 7:**
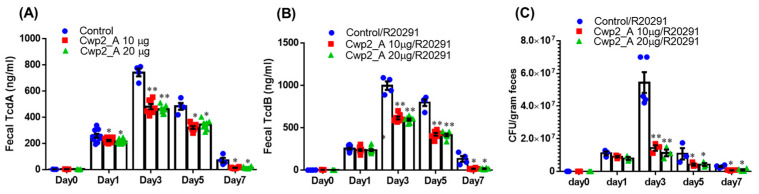
**Immunization of with Cwp2_A reduces *C. difficile* spore and toxin levels in feces of *C. difficile* R20291-infected mice.** *C. difficile* toxin (**A**,**B**) and spore (**C**) levels in feces were determined. Data are presented as the mean ± SEM. (* *p* < 0.05, ** *p* < 0.01 versus control).

Cwp2_A-immunized mice excreted significantly lower amounts of toxin A (Figure 7A) and toxin B (Figure 7B) in their feces compared with the PBS-immunized group. Furthermore, fecal samples from Cwp2_A-immunized mice contained significantly fewer R20291 spores compared with those from the PBS-immunized group (Figure 7C).

### 3.4. Anti-Cwp2_A Serum Inhibits the Binding of C. difficile to HCT8 Cells

**Figure 8:**
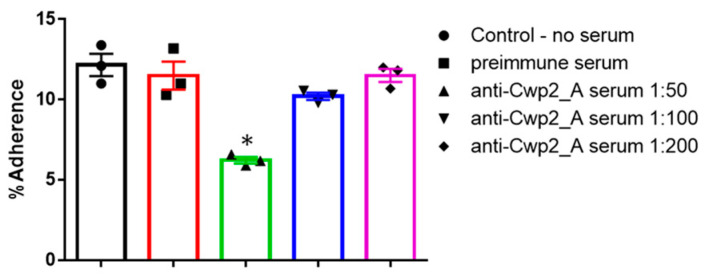
**Anti-Cwp2_A serum inhibits adhesion of *C. difficile* to HCT8 cells.** The adhesion assay was performed as described in the methods. Experiments were independently repeated three times, and data are presented as the mean ± SEM (* *p* < 0.05 vs. treatment with pre-immune serum).

Given that Cwp2_A is predicted to mediate *C. difficile* cell adhesion and that Cwp2_A immunization significantly reduced *C. difficile* spores in infected mice, we hypothesized that anti-Cwp2_A antibodies might inhibit *C. difficile* adhesion to host cells. To test this, we performed an in vitro adhesion assay. When anti-Cwp2_A serum was diluted 1:50 in the cell medium, the adherence rate of *C. difficile* R20291 vegetative cells to HCT8 cells was significantly reduced (6.733 ± 0.5239% vs. 12.17 ± 0.6936%). At a 1:100 dilution, the adherence rate decreased to 10.22 ± 0.223%, though this reduction was not statistically significant (Figure 8).

### 3.5. Immunization of Mice with Cwp2_A Induces Th1- and Th17-Type Immune Responses

**Figure 9:**
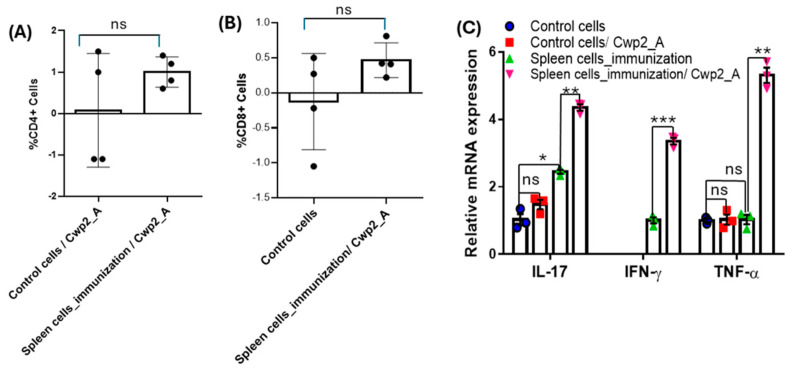
**T cell responses.** Splenocytes from immunized (*n* = 3–4) and unimmunized (*n* = 4) mice were isolated 13 days after the second immunization with Cwp2_A and stimulated with Cwp2_A at 10 µg/mL for 72 h (**A**,**B**) or 6 h (**C**). The proliferative responses of CD4+ (**A**) and CD8+ (**B**) T cells were assayed by staining with appropriate antibodies and were analyzed by flow cytometry. (**C**) IL-17, IFN-γ, and TNF-α expression in the spleen cells was determined by qPCR processing. The y-axis value indicates the expression ratio relative to GAPDH. Data are presented as the mean ± SEM (N = 3, * *p* < 0.05, ** *p* < 0.01, *** *p* < 0.001, ns, not significant).

To evaluate the dominant subset of T cells in the spleen of immunized mice and assess whether the T cells could propagate after re-stimulation with corresponding antigens in vitro, splenocytes were pulsed with Cwp2_A at 10 µg/mL for 72 h. Compared with the unimmunized control group, the percentage of both CD4+ and CD8+ T cells increased in immunized mice, though the results were not statistically significant (Figure 9A,B). Additionally, CD4+ T cells propagated around two times more than CD8+ T cells, indicating a potentially dominant Th cell response in immunized mice in response to Cwp2_A. Because cytokines secreted by the activated T cells are indicators of the type of Th responses, we further assessed the expression of IFN-γ and TNF-α (Th1 response), IL-17 (Th17 response), and IL-4 and IL-5 (Th2 response) by qPCR in the splenocytes stimulated with Cwp2_A at 10 µg/mL for 6 h. The expressions of IL-17, IFN-γ, and TNF-α in splenocytes were all significantly increased after immunization or re-stimulation (Figure 9C); however, the expression of both IL-4 and IL-5 was not detected under experimental conditions. These data support potentially dominant Th1 and Th17 responses induced by Cwp2_A immunization.

## 4. Discussion

Our in silico analysis showed that the functional domain (Cwp2_A) is highly immunogenic and is also conserved. We further evaluated the immunogenicity of Cwp2_A and its protective effect against CDI in mice. Our data showed that the functional domain Cwp2_A is highly immunogenic and conserved. Immunization with Cwp2_A effectively protected mice against CDI and reduced *C. difficile* spore and toxin levels in the feces of mice challenged with *C. difficile* spores. Additionally, anti-Cwp2 serum inhibited the binding of *C. difficile* R20291 vegetative cells to HCT8 cells. Our data suggest that Cwp2 is an important colonization factor for *C. difficile* both in vitro and in vivo and that it alone is a promising vaccine candidate against CDI. These findings are consistent with previous studies reporting that Cwp2_A mediates *C. difficile* adhesion and colonization [25].

*C. difficile* toxin-based vaccine candidates have failed in clinical trials [11,48]. Considering that *C. difficile* is an enteric pathogen, causing high rates of CDI recurrence, effective vaccine candidates should target not only the toxins but also the pathogen colonization to prevent primary and recurrent CDI and disease transmission [40,49]. Furthermore, *C. difficile* vaccine candidates should induce both systematic and intestinal immune antibody responses [49]. To this end, researchers have been actively exploring *C. difficile* surface components as potential vaccine components in combination with toxin-based vaccine candidates. So far, many surface components have been evaluated, which include surface proteins and colonization factors, such as SlpA, Cwp84, Cwp66, FliC, FliD, CdeC, CdeM, cell wall polysaccharides (PS-I, PS-II, PS-III), lipoprotein CD0873, chaperon protein DnaK, heat shock protein GroEL, BclA3, etc., all of which can induce protective immune responses against CDI in animals to different extents; however, none of them have been evaluated in clinical trials [37,50]. Our study demonstrated that Cwp2, which is abundant, rather conserved, and can be easily produced in a large quantity, is highly immunogenic and induces significant protection against CDI as an immunogen, providing a promising option to target *C. difficile* colonization and transmission.

In addition, our data showed that immunization of mice with Cwp2_A induced Th1 (IFN-γ and TNF-α)- and Th17 (IL-17)-type immune responses, but a Th2 (IL-4 and IL-5) response was undetected, as evaluated by qPCR processing based on splenocytes pulsed with antigen for 6 h. This result is somehow unexpected, as aluminum-based adjuvants usually induce an enhanced Th2 immune response but a relatively weak Th1 immune response [51]. We wondered about the potential causes of Cwp2_A-induced preferable Th1 and Th17 immune responses to Th2 response. One major cause could be the aluminum used in the immunizations. Upon writing this manuscript, we realized that the aluminum we used is Imject Alum from Thermo Scientic, which appears to be much less potent than the commonly used aluminum hydroxide (AH) and aluminum phosphate (AP) [52]. Secondly, the antigen restimulation time (6 h) may be too short, as it is difficult to detect secreted cytokines (IL-4, IL-17 and IFN-γ) by ELISA from the splenocytes of the immunized mice pulsed with antigens for 72 h. In the next studies, we will use AH, AP, and liposomes as adjuvants to further evaluate Cwp2_A as a potentially improved vaccine candidate and thoroughly characterize cellular immune responses to the Cwp2_A immunization.

Future studies will also be conducted to identify highly conserved motifs and immune epitopes within the Cwp2_A sequence to enhance the effectiveness of vaccines, potentially in combination with those targeting *C. difficile* toxins and other colonization factors developed by our group [37,39,41]. Additionally, we will aim to explore whether other non-antibody immune responses to Cwp2 contribute to the observed protection against CDI, as it is known that the Cwp2 homolog SlpA plays a role in shaping the host immune response [53,54].

## 5. Conclusions

In conclusion, our report demonstrated for the first time the potential of Cwp2_A as an effective vaccine candidate against CDI in mice, which will be a valuable contribution to our on-going combat against serious infection caused by the multiple-antibiotic resistant *C. difficile*.

## Figures and Tables

**Table 1:** Primers used for real-time PCR processing.

Target	Forward Primer (5′ to 3′)	Reverse Primer (5′ to 3′)
GAPDH	TGCACCACCAACTGCTTAG	GGATGCAGGGATGATGTTC
IFN-γ	AAAGAGATAATCTGGCTCTGC	GCTCTGAGACAATGAACGCT
TNF-α	CCACCACGCTCTTCTGTCTAC	AGGGTCTGGGCCATAGAACT
IL-17	GGAGAAAGCGGATACCAA	TGTGAGGACTACCGAGCC
IL-4	TCTCGAATGTACCAGGAGCCATATC	AGCACCTTGGAAGCCCTACAGA
IL-5	AGCACAGTGGTGAAAGAGACCTT	TCCAATGCATAGCTGGTGATTT

## Data Availability

The data is available from the corresponding author upon reasonable request.

## Supplementary Materials

The following supporting information can be downloaded at: https://www.mdpi.com/article/10.3390/vaccines13010021/s1.
