# Supplementary material for: Cell Wall Protein 2 as a Vaccine Candidate Protects Mice Against Clostridioides difficile Infection
Source: Vaccines (Basel). 2024 Dec 30;13(1):21. doi: 10.3390/vaccines13010021 (PMC11768939; doi:10.3390/vaccines13010021)
Supplement: Supplementary file 1 [file vaccines-13-00021-s001.zip › Supplementary Table 1_08_26_2024.pdf]

Supplementary Material

Supplementary Table S1. *C. difficile* strains used for homology analysis of Cwp2.

Toxinotype	Strain	Ribotype	Database	Accession number/ Barcode	References
A+B+CDT+	LC693	RT871	GenBank	NCXL00000000.1	[1, 2]
	TGH29	ST1	GenBank	JAPKMB000000000.1	[3]
	TGH79	ST11	GenBank	JAPKMA000000000.1	
	TGH35	RT027	GenBank	JAJNGZ000000000	[4]
	TGH64	RT027	GenBank	JAJNHA000000000	
	SMG-20-1235	RT019	Enterobase	CLO_EA5278AA_AS	-
	SMG-21-1765	RT019	Enterobase	CLO_FA2592AA_AS	-
	C00002493	RT 019	Enterobase	CLO_AA4580AA_AS	-
	SIRN_ST-001	RT023	Enterobase	CLO_EA5979AA_AS	-
	CD-16-00530	RT023	Enterobase	CLO_DA8015AA_AS	-
	CD-15-00694	RT023	Enterobase	CLO_DA7541AA_AS	-
	R20291	RT027	GenBank	FN545816.1	[5]
	CD-17-01474	RT027	GenBank	NZ_CP026591.1	[6]
	DSM 27638	RT027	GenBank	CP011846.1	[7]
	CD196	RT027	GenBank	FN538970.1	[8]
	C00002490	RT045	Enterobase	CLO_BA6150AA_AS	-
	CD-16-00514	RT045	Enterobase	CLO_DA7993AA_AS	-
	SIRN_HG-021	RT045	Enterobase	CLO_EA6022AA_AS	-
	C08-686	RT066	Enterobase	CLO_AA9950AA_AS	-
	CD-16-00440	RT066	Enterobase	CLO_DA7971AA_AS	-
	SMG-21-2330	RT066	Enterobase	CLO_FA3269AA_AS	-
	TW11-RT078	RT078	GenBank	CP035499.1	[9]
	R2	RT078	GenBank	CP026614.2	[10]
	M120	RT078	GenBank	FN665653.1	[5]
	C00000224	RT106	Enterobase	CLO_AA4561AA_AS	-
	6058625	RT126	Enterobase	CLO_BA0391AA_AS	-
	CD-16-00082	RT126	Enterobase	CLO_AA7075AA_AS	-
	DSM 29020	RT126	GenBank	CP012325.1	[11]
	C00009691	RT244	Enterobase	CLO_AA0004AA_AS	-
	C00009694	RT244	Enterobase	CLO_AA0003AA_AS	-
	C00009695	RT244	Enterobase	CLO_AA0002AA_AS	-

Supplementary Table S1. Cont.

Toxinotype	Strain	Ribotype	Database	Accession number/ Barcode	References
A+B+CDT-	VPI10463	RT003	Enterobase	CLO_AA6882AA	[12]
	TGH120	RT106	GenBank	JAJNHB000000000	[4]
	CD630	RT012	GenBank	AM180355.1	[13]
	CD-16-00068	RT106	Enterobase	CLO_DA8040AA_AS	-
	TGH91	ST35	GenBank	JAPKLZ000000000.1	[3]
	DH/NAP11/106/ST-42	RT106	Enterobase	CP022524.1	[14]
A-B+CDT-	M68	RT017	GenBank	NC_017175.1	[12]
	DSM 29627	RT017	GenBank	CP016102.1	[11]
	Xy06	RT017	GenBank	NZ_JANFNF000000000.1	Li et al., unpublished
	CF5	RT017	GenBank	NC_017173.1	[15]
	1470	RT017	GenBank	NZ_OEZL00000000.1	[16]
	8864	RT59	GenBank	NZ_OEZE00000000.1	[17]
	SUC36	RT078	GenBank	NZ_OEZZ00000000.1	[18]
	ES130	SLO101	GenBank	NZ_OEZV00000000.1	
	WA151	SKO098	GenBank	NZ_OEZY00000000.1	
	173070	RT015	GenBank	NZ_OEZH00000000.1	
	TGH33	RT017	GenBank	JAJNHC000000000	[4]
	TGH51	RT017	GenBank	NZ_JAJNHD000000000.1	
A-B-CDT-	CD37	RT09	GenBank	NZ_AHJJ00000000.1	[19]
	CCUG37785	ST3	GenBank	NZ_JAGKRT000000000.1	[20]
	Z31	RT009	GenBank	CP013196	[21]
	DSM 28666	RT084	GenBank	CP012321.1	[11]
	DSM 29637	RT032	GenBank	CP016106.1	
	DSM 29688	RT010	GenBank	CP019858.1	
	DSM 28670	SLO 237	GenBank	CP012312	
	DSM 29629	SLO 235	GenBank	CP016104	
	DSM 28669	SLO 091	GenBank	CP012323	
	HGP05	RT125	GenBank	CP103977	[22]
A-B-CDT+	OCD52	RT033	Enterobase	CLO_BA8168AA_AS	-
	IS58	RT033	Enterobase	CLO_AA9965AA_AS	[23]
	RPH0101	RT033	Enterobase	CLO_BA3454AA_AS	-

References for supplementary information

1. Peng, Z.; Liu, S.; Meng, X.; Liang, W.; Xu, Z.; Tang, B.; Wang, Y.; Duan, J.; Fu, C.; Wu, B.; et al. Genome characterization of a novel binary toxin-positive strain of *Clostridium difficile* and comparison with the epidemic 027 and 078 strains. *Gut Pathog.* **2017**, *9*, 42.
2. Li, C.; Harmanus, C.; Zhu, D.; Meng, X.; Wang, S.; Duan, J.; Liu, S.; Fu, C.; Zhou, P.; Liu, R.; et al. Characterization of the virulence of a non-RT027, non-RT078 and binary toxin-positive *Clostridium difficile* strain associated with severe diarrhea. *Emerg. Microbes Infect.* **2018**, *7*, 211.
3. Wickramage, I.; Heuler, J.; Peng, Z.; Alrabaa, S.; Sun, X. Draft Genome Sequences and Genome Characterization of Three Toxigenic and Two Nontoxigenic *Clostridioides difficile* Clinical Isolates from Florida, USA. *Microbiol. Resour. Announc.* **2023**, *12*, e00151-23.
4. Wickramage, I.; Peng, Z.; Chakraborty, S.; Harmanus, C.; Kuijper, E.J.; Alrabaa, S.; Smits, W.K.; Sun, X. The $\textit{vanR}_{Cd}$ mutation 343A>G, resulting in a Thr115Ala substitution, is associated with an elevated minimum inhibitory concentration (MIC) of vancomycin in clostridioides difficile clinical isolates from florida. *Microbiol. Spectr.* **2023**, *11*, e03777-22.
5. He, M.; Sebaihia, M.; Lawley, T.D.; Stabler, R.A.; Dawson, L.F.; Martin, M.J.; Holt, K.E.; Seth-Smith, H.M.; Quail, M.A.; Rance, R.; et al. Evolutionary dynamics of *Clostridium difficile* over short and long time scales. *Proc. Natl. Acad. Sci. USA* **2010**, *107*, 7527–7532.
6. Steglich, M.; Hofmann, J.D.; Helmecke, J.; Sikorski, J.; Spröer, C.; Riedel, T.; Bunk, B.; Overmann, J.; Neumann-Schaal, M.; Nübel, U. Convergent loss of ABC transporter genes from *Clostridioides difficile* genomes is associated with impaired tyrosine uptake and p-cresol production. *Front. Microbiol.* **2018**, *9*, 901.
7. Groß, U.; Brzuszkiewicz, E.; Gunka, K.; Starke, J.; Riedel, T.; Bunk, B.; Spröer, C.; Wetzel, D.; Poehlein, A.; Chibani, C.; et al. Comparative genome and phenotypic analysis of three *Clostridioides difficile* strains isolated from a single patient provide insight into multiple infection of *C. difficile. BMC Genom.* **2018**, *19*, 1.
8. Tasteyre, A.; Barc, M.-C.; Karjalainen, T.; Dodson, P.; Hyde, S.; Bourlioux, P.; Borriello, P. A *Clostridium difficile* gene encoding flagellin. *Microbiology* **2000**, *146*, 957–966.
9. Fatoba, A.J.; Fatoba, D.O.; Babalola, S.O. Pangenome and subtractive genomic analysis of *Clostridioides difficile* reveals putative drug targets. *J. Proteins Proteom.* **2022**, *13*, 247–256.
10. Kumar, A.; Davenport, K.W.; Vuyisich, G.; Kunde, Y.A.; Johnson, S.L.; Chain, P.S.G.; Dichosa, A.E.K.; Rodriguez-Palacios, A. Complete genome sequences of historic *Clostridioides difficile* food-dwelling ribotype 078 strains in Canada identical to that of the historic human clinical strain M120 in the United Kingdom. *Genome Announc.* **2018**, *7*, e00853-18. https://doi.org/10.1128/mra.00853-18.
11. Riedel, T.; Wetzel, D.; Hofmann, J.D.; Plorin, S.P.E.O.; Dannheim, H.; Berges, M.; Zimmermann, O.; Bunk, B.; Schober, I.; Spröer, C.; et al. High metabolic versatility of different toxigenic and non-toxigenic *Clostridioides difficile* isolates. *Int. J. Med. Microbiol.* **2017**, *307*, 311–320.
12. Cairns, M.; Preston, M.D.; Lawley, T.D.; Clark, T.G.; Stabler, R.A.; Wren, B.W. Genomic epidemiology of a protracted hospital outbreak caused by a toxin A-negative *Clostridium difficile* sublineage PCR ribotype 017 strain in London, England. *J. Clin. Microbiol.* **2015**, *53*, 3141–3147.
13. Sebaihia, M.; Wren, B.W.; Mullany, P.; Fairweather, N.F.; Minton, N.; Stabler, R.; Thomson, N.R.; Roberts, A.P.; Cerdeño-Tárraga, A.M.; Wang, H.; et al. The multidrug-resistant human pathogen *Clostridium difficile* has a highly mobile, mosaic genome. *Nat. Genet.* **2006**, *38*, 779–786.
14. Ozer, E.A.; Hauser, A.R.; Gerding, D.N.; Espinosa, R.O.; Hecht, D.W.; Kociolek, L.K. Complete genome sequence of *Clostridioides difficile* epidemic strain DH/NAP11/106/ST-42, isolated from stool from a pediatric patient with diarrhea. *Genome Announc.* **2017**, *5*, e00923-17. https://doi.org/10.1128/genomea.00923-17.

15. Imwattana, K.; Knight, D.R.; Kullin, B.; Collins, D.A.; Putsathit, P.; Kiratisin, P.; Riley, T.V. *Clostridium difficile* ribotype 017–characterization, evolution and epidemiology of the dominant strain in Asia. *Emerg. Microbes Infect.* **2019**, *8*, 796–807.
16. Depitre, C.; Delmee, M.; Avesani, V.; Roels, A.; L'Haridon, R.; Popoff, M.; Corthier, G. Serogroup F strains of *Clostridium difficile* produce toxin B but not toxin A. *J. Med. Microbiol.* **1993**, *38*, 434–441.
17. Soehn, F.; Wagenknecht-Wiesner, A.; Leukel, P.; Kohl, M.; Weidmann, M.; von Eichel-Streiber, C.; Braun, V. Genetic rearrangements in the pathogenicity locus of *Clostridium difficile* strain 8864–implications for transcription, expression and enzymatic activity of toxins A and B. *Mol. Gen. Genet. MGG* **1998**, *258*, 222–232.
18. Janezic, S.; Dingle, K.; Alvin, J.; Accetto, T.; Didelot, X.; Crook, D.W.; Lacy, D.B.; Rupnik, M. Comparative genomics of *Clostridioides difficile* toxinotypes identifies module-based toxin gene evolution. *Microb. Genom.* **2020**, *6*, e000449.
19. Brouwer, M.S.; Warburton, P.J.; Roberts, A.P.; Mullany, P.; Allan, E. Genetic organisation, mobility and predicted functions of genes on integrated, mobile genetic elements in sequenced strains of *Clostridium difficile*. *PLoS ONE* **2011**, *6*, e23014.
20. Wang, S.; Heuler, J.; Wickramage, I.; Sun, X. Genomic and Phenotypic Characterization of the Nontoxigenic *Clostridioides difficile* Strain CCUG37785 and demonstration of its therapeutic potential for the prevention of *C. difficile* Infection. *Microbiol. Spectr.* **2022**, *10*, e01788-21.
21. Pereira, F.L.; Júnior, C.A.O.; Silva, R.O.S.; Dorella, F.A.; Carvalho, A.F.; Almeida, G.M.F.; Leal, C.A.G.; Lobato, F.C.F.; Figueiredo, H.C.P. Complete genome sequence of *Peptoclostridium difficile* strain Z_{31}. *Gut Pathog.* **2016**, *8*, 11.
22. Shivaperumal, N.; Hain-Saunders, N.M.R.; Chang, B.J.; Riley, T.V.; Knight, D.R. Complete genome sequences of evolutionary clade C-III strains of *Clostridioides (Clostridium) difficile* isolated from the environment in Western Australia. *Microbiol. Resour. Announc.* **2023**, *12*, e00239-23.
23. Stubbs, S.; Rupnik, M.; Gibert, M.; Brazier, J.; Duerden, B.; Popoff, M. Production of actin-specific ADP-ribosyltransferase (binary toxin) by strains of *Clostridium difficile*. *FEMS Microbiol. Lett.* **2000**, *186*, 307–312.

Disclaimer/Publisher's Note: The statements, opinions and data contained in all publications are solely those of the individual author(s) and contributor(s) and not of MDPI and/or the editor(s). MDPI and/or the editor(s) disclaim responsibility for any injury to people or property resulting from any ideas, methods, instructions or products referred to in the content.
