# Supplementary figures and images for: Cell Wall Protein 2 as a Vaccine Candidate Protects Mice Against Clostridioides difficile Infection
Source: Vaccines (Basel). 2024 Dec 30;13(1):21. doi: 10.3390/vaccines13010021 (PMC11768939; doi:10.3390/vaccines13010021)

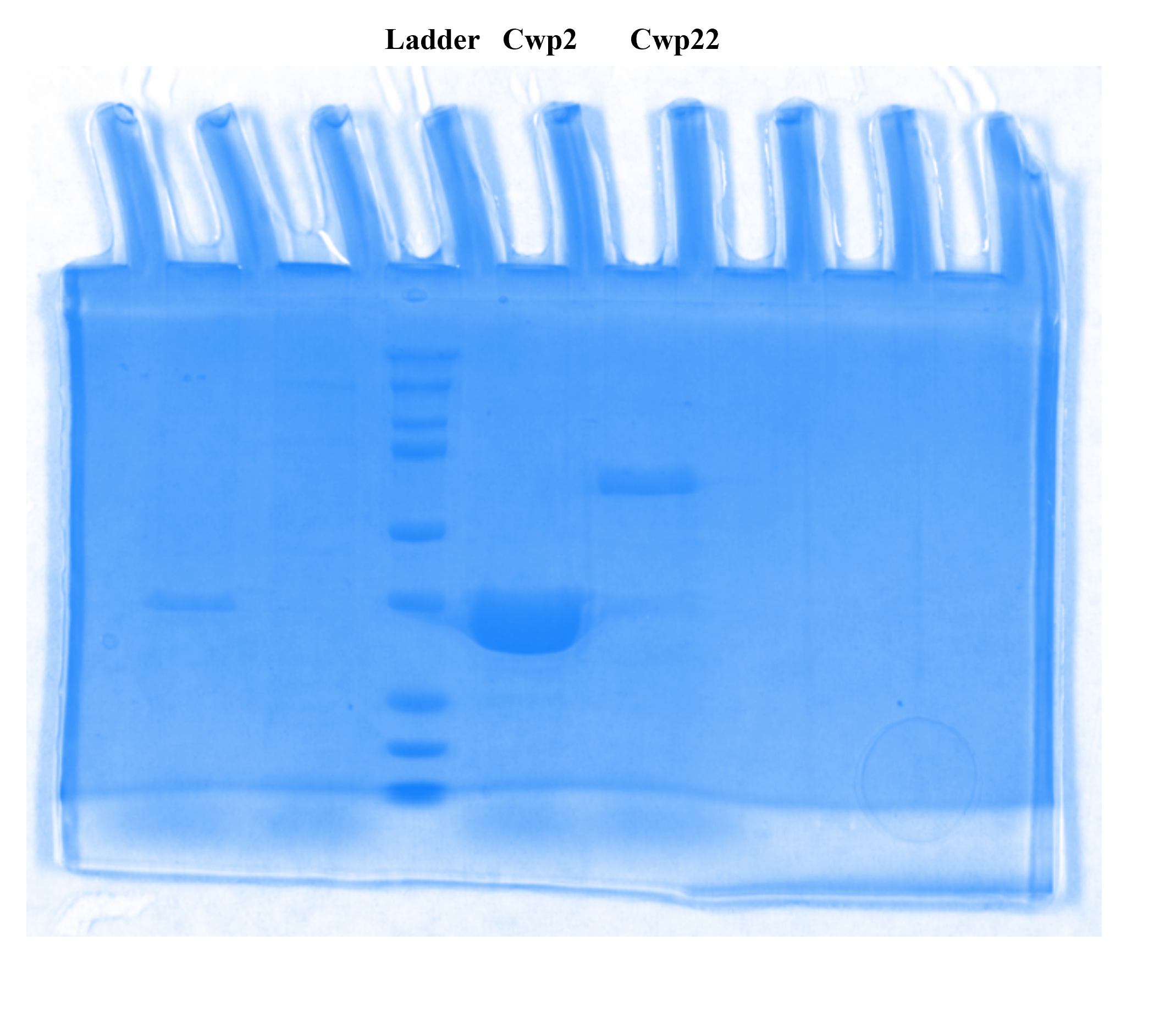

Supplement: Supplementary file 1 [file vaccines-13-00021-s001.zip › Figure 5A uncropped gel.tif]
